# The Potential of Myrtaceae Species for the Phytomanagement of Treated Municipal Wastewater

**DOI:** 10.3390/plants12152844

**Published:** 2023-08-01

**Authors:** Alexandra Meister, María Jesús Gutiérrez-Ginés, Hamish Lowe, Brett Robinson

**Affiliations:** 1 Institute of Environmental Science and Research Ltd., Christchurch 8041, New Zealand; 2 Lowe Environmental Impact, Palmerston North 4410, New Zealand; 3 School of Physical and Chemical Sciences, University of Canterbury, Christchurch 8041, New Zealand

**Keywords:** biowaste, *Kunzea robusta*, land treatment, *Leptospermum scoparium*, native vegetation

## Abstract

The use of native plants in land application systems for treated municipal wastewater (TMW) can contribute to ecological restoration. However, research on the potential of native species to manage the nutrients and contaminants contained in TMW is scarce. At a 10-hectare field site irrigated with TMW at >4000 mm yr^−1^, we investigated the distribution of nutrients and trace elements in the soil–plant system, comparing the New Zealand native Myrtaceae species *Leptosperum scoparium* and *Kunzea robusta* with pasture. The results showed that plant growth did not correlate with TMW irrigation rates. *L. scoparium* and *K. robusta* had higher foliar trace element concentrations than pasture, but these were not correlated with TMW irrigation rates. The pasture accumulated more N and P (68 kg of N ha^−1^ yr^−1^ and 11 kg of P ha^−1^ yr^−1^) than the Myrtaceae species (0.6–17 kg of N ha^−1^ yr^−1^ and 0.06–1.8 kg of P ha^−1^ yr^−1^). Regular harvesting of the pasture would likely remove more N and P from the site than the Myrtaceae species. The results highlight the importance of adjusting TMW application rates to the soil–plant capacity, in which case, native plants could provide ecological or economic value to TMW-irrigated land.

## 1. Introduction

Population growth and economic development are accelerating the production of wastewater [1]. With 65–80% of global freshwater withdrawals being released back into the environment as wastewater [2], the estimated global wastewater production is 359 billion m^3^ yr^−1^ [3]. The management of wastewater is a major social and environmental challenge, with most wastewater, treated or not, being directly released into aquatic environments [4]. Despite the majority of wastewater being treated in high-income countries [5], the discharge of treated municipal wastewater (TMW) can adversely affect water quality [6]. Depending on the level of treatment, TMW contains varying amounts of nutrients and contaminants. When TMW is discharged into waterbodies, these nutrients, primarily N and P, can cause eutrophication and toxic algal blooms [7,8]. While it is possible to reduce the concentration of nutrients and contaminants in TMW to below drinking water standard using advanced treatment options, this greatly increases treatment costs [9]. Alternatively, TMW can be applied to land where the soil–plant system can retain and transform nutrients and contaminants and lead to better environmental and economic outcomes [10].

Treated municipal wastewater is widely used on agricultural land to accelerate the production of crops [11]. However, combining TMW reuse with food production can be restricted by cultural and societal views on waste, as well as industry perceptions and limitations [12]. In such cases, TMW may instead be used for other purposes, such as landscaping or environmental restoration. Examples include the irrigation of golf courses, parks, and gardens, wetland restoration, and the creation of artificial lakes [13,14]. Furthermore, TMW is used to support short rotation coppice [15] and to accelerate the growth of timber species [16]. However, the species used in such systems are often exotic and may disturb local ecosystems, such as *Pinus radiata* (Monterey pine), which has become invasive in large parts of the southern hemisphere [17]. Recent research has focused on the potential of native vegetation as an alternative for the phytomanagement of biosolids [18], municipal wastewater [19], and industrial wastewater [20,21]. Native species are well adapted to the local conditions, promote and preserve native biodiversity, aid with ecological restoration, and can be utilised for the production of high-value native (non-food) products, such as essential oils, timber, and fibre [22,23].

Some native species have the potential to accumulate nutrients in excess of plant requirements, commonly referred to as luxury uptake [24]. Esperschuetz, et al. [25] reported that the New Zealand (NZ) native species *Leptospermum scoparium* (mānuka) and *Kunzea robusta* (kānuka) accumulated three times more N in their stem biomass than *P. radiata*. Furthermore, there is evidence that some NZ native species not only mitigate nutrient losses through plant uptake but distinctively affect the biogeochemical cycling of N. reported significantly lower levels of NO_3_^−^ leaching under *L. scoparium* and *K. robusta* compared to *P. radiata* following the addition of urea to soils. These species can also reduce nitrous oxide (N_2_O) emissions from the soil, likely by inhibiting nitrification and denitrification [25,26]. Replacing *P. radiata* in TMW land application systems with native vegetation may therefore reduce nutrient inputs into ground and surface waters. However, many NZ native species are adapted to low-fertility soils and marginal environments [27]. Therefore, the testing of different species in high-nutrient environments is required to understand candidate species’ potential to manage nutrients and contaminants in TMW land application schemes.

Previous research demonstrated that N fertilisers and biosolids have negligible or positive effects on the biomass production of NZ native plant species [24,28]. Similarly, we showed that NZ native plants can thrive with TMW irrigation at application rates comparable to the annual rainfall [19]. However, the performance of native vegetation on land with the historical application of TMW and irrigation regimes manyfold higher than the annual rainfall is unknown. We aimed to determine the distribution of nutrients and trace elements in soils and plants following the irrigation of TMW at rates up to >4000 mm yr^−1^ in an area that has received TMW for over 30 years. Specifically, we sought to quantify nutrient concentrations in the soil–plant system in *K. robusta* and *L. scoparium* compared to pasture.

## 2. Materials and Methods

### 2.1. Site Description

The study site was located in Levin on the North Island of NZ (40°37′27″ S 175°10′48″ E). The site consisted of sand dunes and plains. It had been used for TMW irrigation from the local wastewater treatment plant onto the *P. radiata* plantation forest since 1986. The wastewater underwent secondary treatment at the Levin Wastewater Treatment Plant, prior to being pumped into a 7-hectare unlined pond at the land application site. From there, the TMW was irrigated onto 40.5 ha of land at an average rate of 4667 mm yr^−1^ [29]. Table 1 shows the concentration of nutrients and contaminants in the TMW and their respective application rates. The sprinkler irrigation system operated overnight and the irrigation of each plot occurred 1–3 times per week, depending on the TMW level in the pond. 

In 2017 and 2018, the *P. radiata* plantation was harvested and ca. 80% of the resulting slash was cleared and piled in windrows. In 2018, 10 ha were substituted with NZ native species, dominated by *Kunzea robusta* (de Lange et Toelken) and *Leptospermum scoparium* (J.R.Forst. et G.Forst). Root trainer-grown seedlings of 0.3–0.5 m in height were planted at a density of 10,000 plants ha^−1^. Following planting, a grass cover, dominated by *Holcus lanatus* L. and *Lolium perenne* L., was sown to prevent the development of further weeds. The soils in the planted areas were well-drained Sandy Recent soils [31]. The median annual temperature at the site was 13.5 °C and the annual rainfall was 1163 mm [32].

### 2.2. Soil and Plant Chemistry

In November 2020, soil and plant samples were collected from a TMW-irrigated plot that had received weed management in the form of manual weeding and herbicide spot spraying. Seventeen specimens each of *K. robusta* and *L. scoparium*, as well as 17 areas of pasture (dominated by *Holcus lanatus* and *Lolium perenne*), were selected at varying distances from central sprinklers. A rain gauge was placed beside each specimen to record the TMW irrigation rates during one run of overnight irrigation. There was no rainfall during this period. The heights of the *K. robusta* and *L. scoparium* specimens were measured with a measuring tape. Foliage was sampled from each specimen by cutting 10 individual branches of varying ages and aspects using secateurs. All branches had a diameter of <5 mm and were cut near the trunk. There were no flowers or fruits on the sampled foliage. To collect pasture samples, a rectangle of 0.2 m × 0.2 m was placed on the ground and all pasture within that rectangle was cut 10 mm above the soil surface. Soil samples were collected from below each sampled specimen, inside the rectangle for pasture and within 0.3 m from the trunk for *K. robusta* and *L. scoparium*. Samples were taken at two depths, using a bucket soil sampler (23 mm diameter) to collect samples from the topsoil (0–10 cm depth) and a soil auger (25 mm diameter) to collect samples from the subsoil (30–45 cm depth). The soils were stored in polyethylene bags, cooled with ice packs, and transported to the lab in insulated containers where they were immediately frozen at −20 °C until analysis.

Soil NO_3_^−^ and exchangeable ammonium (NH_4_^+^) were extracted from fresh soil with 2 M KCl [33]. Colourimetric methods were used to determine the NO_3_^−^-N [34] and NH_4_^+^-N [35] in the extract, using a Cary 100 Bio UV-visible spectrophotometer (Agilent Technologies, Santa Clara, CA, USA). The soil moisture content was determined by drying a subsample of 10–20 g fresh soil at 105 °C for 24 h [33]. The remaining soils were spread on aluminium trays, dried at 40 °C for 4 days, and sieved to ≤2 mm. The plant samples were washed with deionised water before being dried at 60 °C until a constant weight was obtained (4 days). Leaves of the *K. robusta* and *L. scoparium* were separated from the stems. Dried plant leaves and soils were ground with a Rocklabs Bench Top Ring Mill (Scott, Dunedin, New Zealand). A LECO CN828 C and N analyser (LECO, St. Joseph, MI, USA) was used to determine the total C and N contents in the ground soil and plant samples. Soil pH and electrical conductivity (EC) were determined using deionised water in a 1:5 soil:water extract, using a HQ 440d Multi-Parameter Meter with pH probe PHC735 and EC probe CDC40101 (HACH, Loveland, CO, USA).

For the determination of total elemental concentrations, 0.2 g of ground soil or plant material was digested with 5 mL of ultrapure HNO_3_ (69%). Samples were left to pre-digest overnight; they were digested on an ultraWAVE microwave digester (Milestone Srl, Sorisole, Italy) and diluted 21 times with ultrapure water. The element concentrations in the digests were determined by ICP-MS (7500cx, Agilent Technologies, Santa Clara, CA, USA). Certified reference materials were included with the soil and plant digestions (SRM 2710a—Montana I Soil and SRM1573a—Tomato Leaves, National Institute of Standards and Technology (NIST), U.S. Department of Commerce). A total of 0.05 M of Ca(NO_3_)_2_ was used to extract phytoavailable metals from the soil [36]. The extracts were filtered through Whatman No. 42 filter paper and diluted 21 times with ultrapure water. The element concentrations were analysed by ICP-MS (Agilent 7500cx). To determine plant-available P, the soils (air-dried, ≤2 mm) were extracted with 0.5 M of NaHCO_3_ [37]. The P concentration in the extract was determined colourimetrically, using a UV-visible spectrophotometer (Cary 100 Bio).

### 2.3. Statistical Analysis

Data were analysed and visualised using RStudio (R Core Team, 2021). Descriptive statistics included geometric means and standard error ranges for soil and plant variables, due to the range of TMW irrigation rates received by the sampled specimens. Pearson correlation coefficients were determined for correlations between the individual soil and plant variables in each species. The Kruskal–Wallis Test, followed by Dunn’s test, was used to determine significant differences (*p* ≤ 0.05) in the soil and plant parameters between the plant species. Principal component analysis (PCA) was performed using the package ‘factoextra’ [38].

## 3. Results and Discussion

### 3.1. Soil Chemistry

The soil pH was low (Table 2 and Table 3) but still within the typical range for NZ soils, which is 4.1 to 7.4 [39]. Plantation forests and native vegetation typically show lower soil pHs than other land uses in NZ, highlighting the low pH tolerance of these plants [39]. Under pasture, TMW irrigation rates were negatively correlated with soil pH in both the top- and subsoil. This was not observed under *K. robusta* and *L. scoparium*. The negative correlation in this study contrasted with other studies reporting that soil pH increases in TMW irrigation systems [40,41]. Soil acidification may result from increased nitrification with TMW application, which can be a major source of soil acidity [42]. High inputs of N through TMW irrigation likely provided a sufficient substrate for high nitrification rates, and the soil pH was in the optimum range for nitrification (4.5 to 7.4) [43]. Consistently, soil pH was negatively correlated with NO_3_^−^ in the topsoil under pasture (*r* = −0.58, *p* < 0.05). While the measured pH at the site was below the optimum range for the availability of plant nutrients [42], it is unlikely to result in nutrient deficiencies due to the high application rates with TMW irrigation (Table 1).

Total Na concentrations in the topsoil were positively correlated with TMW irrigation under *K. robusta* and pasture, but this was not the case under *L. scoparium*. However, the Na concentrations were only two to three times higher than those measured in a TMW-irrigated silt loam under native vegetation elsewhere in NZ [19], despite the high application rate of Na (Table 1) and the conduction of TMW irrigation for over 30 years. This is consistent with findings by Gutierrez-Gines et al. (2020), whereby Na accumulation is not proportional to Na application and reflects the high mobility of Na in soil (Blume et al., 2016). Total concentrations of Mg, Ca, and K were unaffected by TMW irrigation, in both the top- and subsoil. This was likely due to the low application rate of these elements compared to their total concentrations in the soil. However, Na may have replaced other cations in the soil and there was a negative correlation between extractable Mg and TMW irrigation in the topsoil under *K. robusta* and pasture (Table 2). In the subsoil, extractable Mg was negatively correlated with TMW irrigation under *L. scoparium* and pasture (Table 3). A sodium adsorption ratio (SAR) in the TMW of 4.0 (meq L^−1^)^1/2^, in combination with an EC of 74 mS m^−1^ (Table 1), indicate that ‘slight to moderate’ restrictions on TMW application rates are required [44]. However, the risk of Na-induced clay dispersion negatively affecting infiltration is low, due to the low clay content of approx. 7% in the sandy soil [45,46].

The total C and N concentrations were ca. 10-fold lower than those found in NZ pastoral soils [47]. This is typical for a sandy soil as its ability to store organic matter is low due to the low specific surface area of sand [48]. Soil C and N concentrations did not correlate with TMW irrigation rates. However, total C and total N were strongly positively correlated in the topsoil under all species (*r* = 0.97, *p* < 0.01), which indicates that most of the soil N was present as organic N. This was consistent with about 60% of the TMW-applied N being applied as organic N (Table 1). The concentrations of mineral N were similar to those found in other TMW-irrigated soils [19,49], despite higher application rates in this study. Given the high mobility of NO_3_^−^ in soil [50], soil NO_3_^−^ concentrations likely increased with the onset of TMW irrigation but did not continue to increase over time due to leaching.

The total P concentrations in the soil were 50% of those typically found in NZ pastoral soils [51]. However, the soils tested by McDowell and Condron [51] were mostly silt loams; the lower P concentrations likely derived from a low P retention in the sandy soil at our study site [52]. In contrast, plant-available P in the topsoil, as indicated by Olsen P, was manifold higher (~125 mg kg^−1^) than the suggested target range of 5–50 mg kg^−1^ in NZ soils [53] and was comparable with values reported in market gardens [54]. Olsen P in the topsoil was positively correlated with TMW irrigation under *K. robusta* (*r* = 0.60, *p <* 0.05) and *L. scoparium* (*r* = 0.64, *p* < 0.01). Olsen P is usually correlated with DRP in soil and Olsen P values above 50 mg kg^−1^ can result in high P losses, even via leaching [53,54]. Phosphorus leaching losses of up to 8% of the TMW-applied P were reported in a Recent soil with P application rates equivalent to approx. 50% of those at our study site [40]. Given the high Olsen P content at the site and the relatively high hydraulic conductivity of sandy soils [48], subsurface flow can be expected to contribute to P fluxes into groundwater and the nearby stream [55]. Inputs of P into waterways can lead to eutrophication and the degradation of water quality [56]. Olsen P in the subsoil was significantly higher under pasturethan under *L. scoparium*, which may indicate a higher risk of P losses. It is possible that the root exudation of organic C was higher in pasture than *L. scoparium*, resulting in the increased availability of P [57].

The soil concentrations of trace elements were similar to or below the levels found in NZ pastoral soils [47]. Concentrations of As, Cd, Cr, Cu, Hg, Ni, and Pb in the TMW were below the detection limit (<0.01 mg L^−1^) and within the recommended limits for continuous TMW irrigation [44]. This indicates that the risk of non-essential trace elements accumulating and impairing soil health or plant growth with TMW irrigation is low. However, there was a positive correlation between TMW irrigation rates and As in the topsoil under pasture and in the subsoil under *K. robusta*. In the topsoil, Cd was negatively correlated with TMW irrigation under all species; the same was true for Cu under *L. scoparium* and Pb under *K. robusta*. Soil pH is the major factor controlling the adsorption of trace elements into soil [58]. The solubilities of Cd, Cu, and Pb are high at low soil pH [58], which is likely what resulted in the observed reduction of these trace elements in the topsoil with TMW irrigation. In contrast, As is less mobile at a low soil pH and will likely continue to accumulate with TMW irrigation. However, despite TMW irrigation taking place for over 30 years, As concentrations were in the low range of normal soil levels (0.2–40 mg kg^−1^ [59]).

### 3.2. Plant Growth and Chemistry

**Figure 1:**
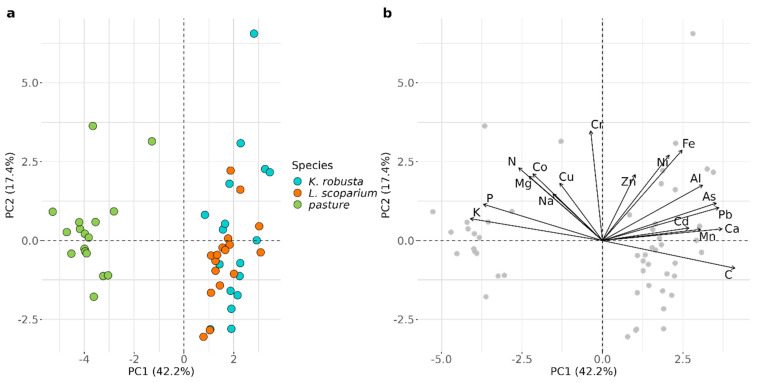
Principal component analysis (PCA) of elemental concentrations in plant foliage; (**a**) score plot and (**b**) loading plot.

There was no correlation between TMW irrigation rates and *K. robusta* and *L. scoparium* height. Similarly, there was no correlation between TMW irrigation rates and pasture biomass (Table 4). This is in contrast with studies by Gutierrez-Gines, et al. [60] and Meister, et al. [19], who found positive growth responses of pasture and native vegetation to TMW irrigation. However, in contrast to these studies, the current site received higher rates of TMW irrigation and had done so for more than 30 years. It is likely that the plants were not limited by nutrient availability and, therefore, did not show a positive growth response to further nutrient addition, particularly because the average application rates of the major plant nutrients, such as N, P, and K, exceeded plant requirements [44]. However, the concentrations of N and P in pasture did not differ from the average NZ values reported by Reiser, et al. [47]. In contrast, the concentrations of N, P, and K in *K. robusta* and *L. scoparium* were higher than in other unamended soils [61]. This is consistent with the luxury uptake of nutrients previously observed in native species [24]. The chemical composition of *K. robusta* and *L. scoparium* differed from the pasture, which contained higher concentrations of macronutrients and lower concentrations of micronutrients than the Myrtaceae species (Figure 1). However, non-essential trace elements, that may have had a negative effect on plant growth, were similar to or lower than those found elsewhere in *L. scoparium* [62], *K. robusta* [63], and pasture [47], which is consistent with the low application rates of these elements (Table 1).

**Figure 2:**
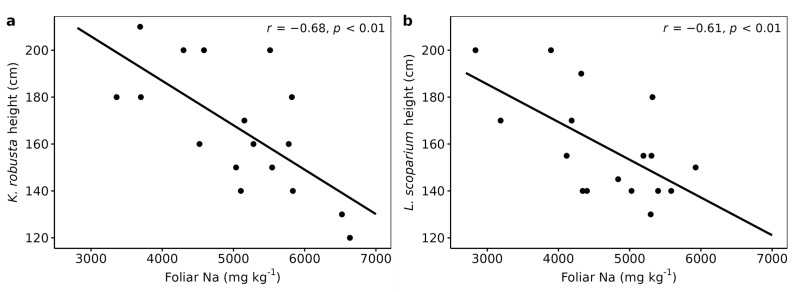
Foliar Na versus plant height; (**a**) *Kunzea robusta* and (**b**) *Leptospermum scoparium*. R values are Pearson correlation coefficients.

There was a significant negative correlation between the heights of *K. robusta* and *L. scoparium* and their foliar Na concentrations (Table 4, Figure 2). The foliar concentration of Na was two to three times higher than those reported in naturally established stands of *L. scoparium* [62]. Similarly, they were up to five and eight times higher than in *K. robusta* and *L. scoparium* grown in pot experiments, respectively [18,64]. The pasture Na concentrations were 37% higher than those reported by Gutierrez-Gines, et al. [60] in pasture grown on a Fluvial Recent soil with TMW irrigation at 1672 mm yr^−1^. Consistent with their study, this shows that pasture Na uptake increases with increased Na application rates. While the TMW contained high concentrations of Na (Table 1), it is possible that sea spray also increased the Na concentrations in plants. The site is located about 1 km east of the coast and strong westerly winds are common in this area, increasing sea spray [65]. It has been demonstrated that sea spray can affect pasture Na concentrations up to 50 km inland [66]. Although Na is not an essential element for most terrestrial plants and can be directly toxic, with woody species being most susceptible [67,68], there were no visual signs indicating such toxicity in *K. robusta* and *L. scoparium*.

### 3.3. Phytomanagement Potential

The concentrations of plant nutrients in both the top- and subsoil did not differ between plant species (Table 4). This indicates that TMW irrigation at >4000 mm yr^−1^ and relative nutrient application rates (Table 1) exceeded plant requirements and uptake capacity. The uptake of N by pasture was 204 kg of N ha^−1^, equivalent to 68 kg of N ha^−1^ yr^−1^ during the three years of growth or 3.0% of the TMW-applied N. Similarly, the uptake of P by pasture was 32 kg of P ha^−1^. This was equivalent to 11 kg of P ha^−1^ yr^−1^ or 3.6% of the TMW-applied P. Pasture could therefore likely only remove a fraction of the applied N and P, even if it were harvested regularly. This is not consistent with uptake rates reported for cut-and-carry pasture, where regular harvesting can remove large proportions of the nutrients applied to land with wastewater [60,69]. Furthermore, the elemental composition of the TMW-irrigated pasture might make it unsuitable for stock fodder, with K concentrations exceeding the maximum tolerable levels [70]. Excess dietary K intake in dairy cows is associated with an increased predisposition to milk fever [71].

Marden and Lambie [72] found that, regarding *L. scoparium*, annual rates of biomass production ranged from 27 to 785 kg ha^−1^ yr^−1^ at a plant density of 784–1242 plants ha^−1^. A similar plant density was measured during sampling and represents a survival rate of 8–12% of the initial plantings [73]. At such biomass production rates, and assuming that the measured foliage N and P concentrations are representative for all of the aboveground biomass, this would be equivalent to accumulation rates of 0.6 to 17 kg of N ha^−1^ yr^−1^ and 0.06 to 1.8 kg of P ha^−1^ yr^−1^. For both elements, these uptake rates are manifold lower than the amount of N and P applied with TMW. The N uptake aligns with that reported in 25-year-old stands of *L. scoparium* and *K. robusta*, which are 104 and 22 kg of N ha^−1^, respectively [74]. These results indicate that pasture has a better potential to extract N and P than the Myrtaceae species. If native plant survival rates were as high as 80%, as reported at another TMW irrigation site in NZ [19], N and P accumulation rates may be eight-fold higher; yet, they would be <50% of the mass that is accumulated by pasture.

Despite the difference in the N and P uptakes between the tested species, there were no differences in soil nutrients between the Myrtaceae species and pasture (Table 2 and Table 3). This indicates that excess N that is not taken up by plants will be lost from the soil. Potential pathways are through the leaching of NO_3_^−^ and organic N or through gaseous N emissions, following volatilization and denitrification [52]. While previous studies showed different effects of *L. scoparium* and *K. robusta* on soil N concentrations, as well as losses compared to other species [25,26], any such effects were likely outweighed by high N application rates at the study site. Therefore, immobile nutrients, such as NO_3_^−^, will readily leach from the soil into groundwater, as well as the nearby stream.

In contrast to plant macronutrients, the concentrations of some micronutrients and non-essential trace elements (As, Cd, Mn, Pb, and Zn) were significantly higher in the Myrtaceae species than in the pasture. Although trace elements were applied at negligible rates (Table 1), this indicates that *K. robusta* and *L. scoparium* may be suitable to manage trace elements from other land-applied biowastes that have higher concentrations thereof, such as biosolids or industrial wastewater [20,28].

## 4. Conclusions

The application of TMW at a rate of >4000 mm yr^−1^ did not directly impair the growth of *L. scoparium*, *K. robusta*, and pasture, as measured by plant height and biomass after three years of irrigation. However, the application rates of nutrients were higher than what the plants, both the pasture and Myrtaceae species, could uptake or manage, which implies a loss of nutrients into the ground and surface waters, as well as the atmosphere. *K. robusta* and *L. scoparium* had higher concentrations of non-essential trace elements, indicating that they are better suited to manage these than pasture. In contrast, the uptake rates of N and P by the Myrtaceae species were estimated to be manifold lower than in pasture. However, to remove nutrients and contaminants from the site and prevent them from re-entering the soil, plant biomass would need to be harvested, for example, to extract high-value essential oils from *K. robusta* and *L. scoparium*. Pasture, on the other hand, could be used as cut-and-carry fodder; but, its K concentration would need to be monitored to not exceed stock fodder limits. This study highlights that TMW application rates must be adjusted to plant requirements and phytoremediation potential to avoid the losses of nutrients and contaminants from TMW-irrigated land. Future research should quantify nutrient losses, occurring through leaching and gaseous emissions, for example, from TMW-irrigated native vegetation. This will allow for the selection of suitable native species for the management of TMW to provide ecological and biodiversity benefits from TMW-irrigated land.

## Figures and Tables

**Table 1:** Concentration of nutrients and contaminants in treated municipal wastewater (TMW) and relative application rates upon TMW irrigation of 4667 mm yr^−1^.

Parameter	Concentration in TMW (mg L^−1^) ^a^	Application (kg ha^−1^ yr^−1^)
NO_3_^−^-N	11 ± 1.2	513
NH_4_^+^-N	8.0 ± 1.1	373
Total N	48 ± 3.0	2240
PO_4_^3−^-P	1.2 ± 0.19	56
Total P	6.6 ± 0.91	308
Ca	12 ± 0.19	560
Mg	3.2 ± 0.13	149
K	25 ± 1.9	1167
Na	61 ± 3.2	2847
B	163 ± 12 (μg L^−1^)	7.6
As, Cd, Cr, Cu, Hg, Ni, Pb	<0.01	<0.47
Total suspended solids (TSS)	13 ± 6.3	607
Electrical conductivity (EC)	74 ± 5.1 (mS m^−1^)	-
Sodium adsorption ratio (SAR) ^b^	4.0 ((meq L^−1^)^1/2^)	-

^a^ Mean ± standard error (*n* = 4–145). Values are in mg L^−1^, unless otherwise indicated. ^b^ SAR expresses the concentration of Na relative to Ca and Mg, calculated after Ayers and Westcot [30].

**Table 2:** Treated municipal wastewater (TMW) irrigation and soil chemistry at a 0–10 cm depth under *Kunzea robusta*, *Leptospermum scoparium*, and pasture.

0–10 cm	*K. robusta*	*L. scoparium*	Pasture
Irrigation (mm day^−1^)	11 (2.5–50)	10 (2.4–43)	12 (2.6–81)
pH	4.7 (4.4–5.0)	4.7 (4.4–5.0)	4.8 (4.4–5.2) **[−S]**
EC (dS m^−1^)	149 (83–265)	116 (54–250)	118 (73–235)
Total C (%)	5.5 (3.4–8.7)	4.9 (2.6–9.0)	5.4 (3.5–10)
Total N (%)	0.27 (0.17–0.33)	0.25 (0.13–0.49)	0.28 (0.18–0.58)
NO_3_^−^-N	7.5 (2.5–23)	7.0 (1.9–26)	4.9 (1.8–16) [+S *]
NH_4_^+^-N	19 (11–33)	16 (9. 1–30)	16 (9.7–30)
Total P	579 (500–671)	577 (491–678)	535 (456–634)
Olsen P	131 (105–163) **[+S]**	121 (66–222) [+S *]	122 (91–214)
Total Na	734 (616–876) **[+S]**	803 (689–936)	708 (589–878) [+S *]
Total K	2584 (1999–3088)	2506 (2080–3019)	2278 (2008–2736)
Total Ca	7208 (6643–7822)	7476 (6988–7997)	7233 (6746–7834)
Total Mg	2570 (2325–2842) ^ab^	2580 (2299–2896) ^a^	2409 (2265–2631) ^b^
Extractable Mg	129 (62–269) [−S *]	158 (87–287)	99 (37–235) **[−S]**
Total As	2.3 (1.9–2.6) ^a^	2.3 (2.0–2.5) ^ab^	2.0 (1.9–2.3) [+S *] ^b^
Total Cd (µg kg^−1^)	8.3 (5.6–12.3) [−S *] ^a^	6.5 (4.8–8.8) **[−S]** ^ab^	5.5 (3.9–8.2) **[−S]** ^b^
Total Cu	5.0 (4.1–5.9)	4.7 (3.7–5.8) [−S *]	4.6 (3.7–5.8)
Total Pb	4.2 (3.4–5.3) **[−S]**	4.0 (3.6–4.5)	3.9 (3.5–4.8)

Values are geometric means and standard deviation ranges (*n* = 17). Values are in mg kg^−1^, unless otherwise indicated. Variables that were significantly correlated (+positive/−negative) with TMW irrigation rates are indicated in bold in square brackets; S: *p* ≤ 0.05, S *: *p* ≤ 0.01. Different superscript letters indicate significant differences between species at *p* ≤ 0.05.

**Table 3:** Soil chemistry at a 30–45 cm depth under *Kunzea robusta*, *Leptospermum scoparium*, and pasture.

30–45 cm	*K. robusta*	*L. scoparium*	Pasture
pH	5.3 (4.9–5.7)	5.5 (5.0–6.1)	5.3 (4.8–5.8) **[−S]**
EC (dS m^−1^)	30 (20–47)	27 (19–39)	22 (17–32) **[+S]**
Total C (%)	0.7 (0.5–1.0)	0.6 (0.5–0.8)	0.7 (0.5–0.9)
Total N (%)	<0.05	<0.05	<0.05
NO_3_^−^-N	2.0 (0.6–6.4)	1.9 (0.6–5.9)	1.1 (0.3–3.9)
NH_4_^+^-N	4.6 (2.5–8.5)	4.2 (2.3–7.5)	4.0 (2.0–7.2)
Total P	388 (307–490)	372 (299–464)	417 (325–524)
Olsen P	39 (22–66) ^ab^	28 (12–62) ^b^	50 (27–110) ^a^
Total Na	734 (658–818)	712 (586–866)	680 (570–817)
Total K	2121 (1683–2676)	2416 (2163–2700)	2118 (1640–2423)
Total Ca	7418 (6771–8126)	7726 (7296–8182)	7562 (6487–8277)
Total Mg	2880 (2584–3209)	3058 (2821–3315)	2882 (2407–3198)
Extractable Mg	30 (18–52)	32 (19–54) **[−S]**	26 (14–44) **[−S]**
Total As	2.5 (2.2–2.9) **[+S]**	2.6 (2.4–2.9)	2.6 (2.2–3.0)
Total Cd (µg kg^−1^)	5.2 (3.5–7.9)	6.5 (5.3–4.1)	5.1 (3.5–7.7)
Total Cu	3.7 (3.4–3.9)	3.7 (3.3–4.1)	3.7 (3.2–4.0)
Total Pb	3.7 (3.4–4.2)	3.8 (3.6–4.1)	3.8 (3.4–4.2)

Values are geometric means and standard deviation ranges (*n* = 17). Values are in mg kg^−1^, unless otherwise indicated. Variables that were significantly correlated (+positive/−negative) with TMW irrigation rates (Table 2) are indicated in bold in square brackets; S: *p* ≤ 0.05, S. Different superscript letters indicate significant differences between species at *p* ≤ 0.05.

**Table 4:** Plant height (*Kunzea robusta* and *Leptospermum scoparium*), biomass (pasture), and elemental concentrations in plant foliage. Biomass N and P in pasture were extrapolated from a harvested area of 0.04 m^2^.

Parameter	*Kunzea robusta*	*Leptospermum scoparium*	Pasture
Plant height (cm)	164 (140–194)	157 (137–180)	n.d.
Plant biomass (g m^−2^)	-	-	725 (525–835)
C (%)	51 (51–52) ^a^	52 (52–53) ^a^	43 (42–44) ^b^
N (%)	2.0 (1.7–2.4) ^b^	2.2 (1.9–2.5) ^b^	2.8 (2.2–3.2) [+S **] ^a^
Biomass N (kg ha^−1^)	-	-	204 (135–208) **[+S]**
K (%)	0.6 (0.5–0.7) ^b^	0.6 (0.5–0.7) **[−S]** ^b^	3.7 (3.0–4.4) ^a^
P (mg kg^−1^)	2695 (2272–3196) **[+S]** ^b^	2398 (2009–2864) ^b^	4386 (3926–5314) ^a^
Biomass P (kg ha^−1^)	-	-	32 (24–44)
Mg (mg kg^−1^)	1454 (1167–1746) ^b^	1614 (1371–1827) ^ab^	1884 (1550–2260) ^a^
Na (mg kg^−1^)	4991 (4097–6082)	4575 (3745–5588) **[+S]**	5480 (3229–7013) [+S **]
Zn (mg kg^−1^)	51 (39–66) ^a^	30 (23–39) **[−S]** ^b^	35 (28–54) [+S *] ^b^
Mn (mg kg^−1^)	1341 (907–1983) ^a^	651 (411–1032) **[−S]** ^b^	177 (108–338) **[−S]** ^c^
Cu (mg kg^−1^)	3.1 (1.9–5.0)	3.3 (2.4–4.5) [−S *]	4.2 (3.1–6.1)
Cr (mg kg^−1^)	0.43 (0.22–0.84) **[+S]** ^ab^	0.33 (0.18–0.59) **[+S]** ^b^	0.57 (0.42–1.3) ^a^
As (µg kg^−1^)	57 (43–77) **[+S]** ^a^	67 (49–91) ^a^	22 (14–30) ^b^
Cd (µg kg^−1^)	17 (10–29) ^a^	12 (7.4–19) ^a^	2.4 (0.9–4.1) ^b^
Pb (µg kg^−1^)	82 (55–121) [+S *] ^a^	83 (54–127) ^a^	14 (5.3–21) **[+S]** ^b^

Values are geometric means and standard deviation ranges (*n* = 17). Variables that were significantly correlated with TMW irrigation rates (Table 1, Table 2, Table 3, Table 4) are indicated in bold and square brackets; S: *p* ≤ 0.05, S *: *p* ≤ 0.01, S **: *p* ≤ 0.001. Different superscript letters indicate significant differences between species at *p* ≤ 0.05.

## Data Availability

The data presented in this study are available on request from the corresponding author.
